# Therapeutics of the Throat

**Published:** 1888-05

**Authors:** 


### THERAPEUTICS OF THE THROAT.

### CARBO ANIMALIS.

This drug ought not to be blindly identified with Carbo vegetabilis, as has been done by some. Compare the following distinctive symptoms with those of Carbo veg., and all thought of *identity* will vanish:

*Objective.*

Pharynx full of mucus, hanging in long tough strings, requiring hawking all morning, till noon. (*Carbo veg., loose masses, easy hawking.*)

Ptyalism during sleep.

Aphonia *at night.* (*Carbo veg. morning.*)

Tongue *immovable*, speech drawling, slow. (Scirrhus.)

Glands (salivary and cervical) *indurated*, STONY.

COPPER-COLORED SPOTS ON SKIN.

Thyroid swollen (in young subjects).

*Subjective.*

Mucus in throat, *keeps one awake all night*, from fear of suffocation, which threatens as soon as the eyes close. C. Hg.

*Causes, aggravations, and aversions.*

Cancerous and syphilitic cases.

Scrofula in young people.

Scurvy.

Morning (hoarseness; hawking of mucus).

Noon (debility).
Evening (hoarseness followed by *aphonia*).
Night (fear of dark; fear of shaking).
Lying on side.
Open air; exertion; tobacco smoke (*Ign.*).
Lactation; sudden prostration, as babe gets through nursing (*Cocculus, Oleander*).
Menstruation (legs and thighs give out).
FISH (good or bad).
Bad weather (scars and cicatrices open; stinging).
Shaving (toothache from).
Aversion to fats.

*Ameliorations and desires.*

Lying on *back.*
Light.
Craving for fresh things; acids; pickles.

*Concomitants.*

Presbyopia (Carbo veg., *myopia*).
Swollen glands *painful.*
Dyspeptic symptoms, as in *Carbo veg.*

*Remarks and comparisons.*

Effects deeper organic changes, as in glands, skin, joints, and other inferior structures, than *Carbo veg.*, and is preferable in glandular affections, especially cancerous and syphilitic, and in scurvy. The patient feels as if homesick; is easily frightened (as by the dark), and the moods are very changeable. The head is easily confused, so that one is not sure if one has slept the previous night or not; SOUNDS ARE NOT EASILY LOCATED IN THE PROPER DIRECTION, the complexion is earthy and cachectic; *the sweat is* YELLOW, *fetid, and profuse,* the feet are extremely cold, the nervous system weak and easily congested, as shown in the color of cancers and other tumors (compare *Lach., Secale, Puls.*); there is *marked and sudden prostration from nursing the baby, just as it finishes; menstruation* is attended with prostration and *weakness of thighs.* Scars and cicatrices sting and open, especially in bad weather.

Carbo animalis is complimented by *Calc. phos.*, and may be compared with *Carbo veg., Calc. c., Caust., Ignat., Zinc, Graph., Badiaga, Brom.*, and the cognates of *Carbo veg.*

It is antidoted by *Ars., Camph., Nux vom., Wine.*

It antidotes bad effects of Quinine. (Suppression of intermittents.

### CARBO VEGETABILIS.

*Objective.*

Palate swollen; blistered; *aphthous.*
Uvula swollen, inflamed.
Tonsils swollen; gangrenous; ichorous discharge.
PHARYNX DRY, GRANULAR (as in measles).
" bluish tint, venous capillary dilatation; ecchymosis.
" full of phlegm, easily hawked up with flow of saliva.
Larynx ulcerated (chronic).
" visible surface, vocal cords, etc., swollen, dingy-purple.
Cervical glands swollen, especially toward nape.
Smell from mouth fetid; *cadaverous.*
Rattling, choking, gagging; *easy hawking of small masses of green or yellow mucus.*
HOARSENESS; APHONIA; THE ROUGH VOICE FAILS ON EFFORT.
Breath cold; Cheyne-Stokes respiration.

*Subjective.*

*Aching back part of palate.*
Biting and burning in back part of fauces, as at the beginning of a cold, but more violent.
Scraping and tingling, *alleviated only for a short time* by clearing the throat.
Small burning spots in throat.
*Roughness, rawness, and dryness, "as if dried with blotting paper;" worse when swallowing.*
ITCHING, extending to ears, with desire to swallow, which act relieves.
Fullness and pressure as if obstructed.
Cramping *as if constricted*, hindering swallowing, but without pain.
*Speaking aloud requires an effort;* seeming as if out of breath.
*Reading aloud fatigues greatly.*
Fauces and posterior nares feel sore on blowing the nose, swallowing, or coughing.
Frequent irritation as if to cough (*Phos.*).
Tearing in muscles of throat and neck (*Rhus*).
Bitterness of palate, with dryness of tongue.

*Causes, aggravations, and aversions.*

Singing, reading aloud, and speaking (*Caust.*, *Ferr.*, *Phos.*, *Arum.*).
Swallowing (dryness; constriction; soreness).
Morning (aphonia, dryness) (*Carbo an.*, *Phos.*, EVENING).
NOON (prostration, drowsiness) (*Sulphur*).
Afternoon (4 to 6, mental anguish) (*Lycop.*).
Evening (hoarseness, *especially in warm damp air*) (*Caust.*, *morning, cold, dry air*).
Night (fright, fear of ghosts) (*Carbo an.*).
Meal, during (dryness, soreness).
" after (eructations, painful hiccough).
Blowing nose, swallowing, coughing (soreness of pharynx, etc.).
Catarrh (loss of voice).
Tuberculosis (laryngitis, hectic).
Measles (ailments of pharynx, ears, eyes, nose, and skin).
Scarlatina; diphtheria (gangrene of tonsils, etc.).
Cholera (collapse, aphonia, cold breath).
Yellow fever (fetid breath, blue tongue, collapse [acts as prophylactic]).
Walking in open air or cold halls, etc.
Warm, close, damp air (cough; hectic; intermittents).
Changes of weather. West winds.
Sexual excesses, onanism, etc.
Clearing throat (ptyalism, lachrymation).
Acute diseases (prostration).
Abuse of Mercury or Cinchona.
Lying down (anxiety) (numbness of limbs).
Washing and bathing.
Heat of bed; overheating in general.
Aversion to meats, fats, coffee, milk.
Ailments from putrid meats (*Carbo an.*, *fish*).
Aversion to exercise; ailments from overlifting; from dislocations.

*Ameliorations and desires.*

Swallowing (itching in pharynx and ears).
Clearing throat; short cough (temporary).
Eructations and passage of flatus (temporary).
FANNING STRONGLY.
Sitting up and moving (choking).
Warm, dry air; light; *leaning or lounging;* coffee.
Desire for sweets and salts; for sauerkraut.

*Concomitants.*

Tongue white; white with red edges; yellow-brown; purple; aphthous; dry; raw; [stinging, tearing, sensitive, *right side.*]

Tongue heavy and inflexible, hindering speech.

" dry as if from lime, or too much wine.

Gums recede from teeth; bleed easily; sore; pustular; aphthous.

Œsophagus feels as if contracted.

" hiccough felt in.

Burning in stomach, with a creeping sensation upward.

ERUCTATIONS, GENERALLY FŒTID OR CADAVEROUS.

STOMACH AND ABDOMEN INFLATED AS IF TO BURSTING; FŒTID FLATUS.

Cough in occasional paroxysms; followed by severe *glowing burning* in chest.

Headache, *sensation as if the removed hat were still on.*

Itching around eyelids (as in measles) (Puls., Euphras.).

Ears feel as if curiously stopped by a finger, or by "little bags of sand laid against them."

Itching around nostrils (Arum.).

Habitual nosebleed; preceded and followed by great pallor of face.

Grayish yellow complexion; great pallor (Ars.).

Emaciation, especially of face.

Depression not preceded by erethism or excitement.

Varices (*Lycopod.*).

Myopia (*Carbo an.*, PRESBYOPIA).

COLDNESS OF KNEES, *even waking one at night* (Menyanthes, *cold below knees*).

*General dullness and languor, not restless* (Bry.).

*Rancid stomach, the most innocent food will disagree.*

Absence of wax in ear.

Nose stopped up with thick yellow matter (*Puls.*).

*Remarks.*

ALL EFFORTS GENERALLY RESULT IN FAILURE, as, trying to sing, speak, swallow, breathe freely, clear the throat; to rest by lying down, to exercise for strength, to exert sexual functions, to digest food, to recover strength after acute disease; trying to hear, to look steadily at objects, to resist the weather, etc.: in all these particular attempts very poor success is met, and *Carbo veg.* will help them all.

Its general *keynotes* seem to be the following :
*Languor without restlessness.*
*Sudden debility,* AT NOON.
*Rancid or fœtid belching, with rumbling and much flatus.*
GLOWING BURNING *in chest, after cough.*
*Sensation as if the removed hat were still on.*
COLDNESS OF KNEES.
*Desire to be strongly fanned.*
*The rough voice fails on effort.* [Complete aphonia (*Caust.*).]
*Sexual debility.*
*Varicose states of veins and capillaries.*
*Is prophylactic against yellow fever.*
Has cured *after pains located in shinbone.*
Relations : Complements the action of *Phos.*, and is complemented by *Kali-carb.*
Precedes well : *Ars., Cinchona, Dros., Kali. c., Merc., Phos. ac.*
Follows well : *Phos., Kali. c., Lach., Sep., Nux vom., Sulph., Tabac., Verat. alb.*
Incompatibles : *Carbo an., Causticum.*
Compares well with all the above, and also with *Secale, Silic., Bry., Lycopod., Eupat. perf., Calc. c., Puls., Rhus, Nitr. ac., Bell., Antim tart., Ferrum, Phos.*
It antidotes *Cinchona, Lach., Merc.,* and is antidoted by *Ferrum, Ars., Camp., Coff., Lach., Spir. nitr., Dulc.*

E. CRANCH.

---

### SEPIA.

### OBJECTIVE SYMPTOMS.

Fauces *red,* throat *bright red* and dry, redness of both sides of throat. *Left tonsil* much swollen, covered with pustules. Inflammation, great swelling, and suppuration of left tonsil. *Constant accumulation of mucus in throat, which almost suffocates.* Dryness and roughness of tongue and palate. Left side of throat and tongue, and corner of the mouth covered with vesicles. Swelling of the cervical glands, submaxillary glands swollen; left submaxillary gland and tonsil very much swollen. Slight swelling of the right parotid gland in the evening. Hawking up phlegm and bloody mucus in the morning.

### SUBJECTIVE SYMPTOMS.

Throat dry and hot with paroxysms of pain attended by lachrymation ; relieved by detaching mucus, which has to be swallowed. *Raw feeling in the posterior nares,* with *dryness and rigidity in the throat below;* throat so dry as to seem like a board, as though the opposing parts would not blend in swallowing. Constant dryness and contracted sensation in throat, in evening before going to sleep. Dryness in throat not relieved by drinking. *Dryness in posterior nares, yet much mucus in the mouth* with involuntary urgings to swallow. *Sensation of a plug in throat;* dryness with sense of thickness in throat. Sore pain in throat when swallowing, immediately after a meal. Feeling like cramp in throat at inner side of cervical vertebræ. Trapezius muscles very sore and sensitive to the touch. Painful contraction and pressure in throat. Pressive pain in upper right side of throat; pressure in throat as if choked with something which would not go down, pressure as from a plug which it seemed he must swallow. Pressure in throat toward the back when swallowing food and drink. Pressure in throat, when dressed ever so loosely. Pressure in region of the tonsils as if the cravat were tied too tightly. Pressure and cutting in throat when swallowing, with a coating of mucus in the throat. *When attempting to hawk the mucus,* the *pressure and cutting are aggravated,* with sensation as if throat were cut with shears, followed by bleeding. Great accumulation of mucus in throat during night. Burning heat in throat with fullness and pressure in head; smarting, then cutting; at times also pressing sensation in left side of throat; raw feeling, accumulation of much phlegm. Throat *feels as if it had been skinned;* pinching in throat from larynx upward; slight creeping in throat; scraping, sticking pain in uvula; very sensitive on swallowing, with shaking chill and collection of mucus, which cannot be loosened. Pain prevented swallowing. Numb feeling in right tonsil. Smarting and burning on posterior portion of fauces and palate, as from violent coryza. Muscles of deglutition seem paralyzed.

### LARYNX AND COUGH.

Frequent pressure in the larynx. Feeling of dryness in larynx. Accumulation of much mucus in larynx, difficult to cough, but easy to swallow.

Sudden *hoarseness.*

*Hoarseness* and fluent coryza.

*Hoarseness,* she is unable to sing high notes. *Hoarseness,* unable to utter a loud word. *Hoarseness with dry cough,* from titillation in the throat. Cough caused by titillation in the larynx without expectoration. Short and hacking cough when going to bed.

Cough, frequently dry whooping and choking,* with pain in the pit of the stomach, and scraping, raw, sore pain in the larynx, not felt when swallowing food; the cough does not rouse her from sleep, but after waking is violent and continuous; sometimes rattling in the trachea resulting in mucous expectoration.

Cough in evening in bed, with vomiting of bile; cough with nausea and retching.

### AGGRAVATIONS AND CONDITIONS.

*Deglutition.*—Stitching pain in the throat, pressure and cutting in throat; pressure and cutting toward the back, when swallowing food and drink. *Pressure in throat as if cravat were tied too tightly.*

*Empty deglutition.*—Stitching, scratching, and sore pain in the fauces.

*Hawking.*—Pressure and cutting in throat, roughness and burning in fauces.

*Smoking.*—*Contracts the fauces.*

*After a meal.*—Immediately, feeling like cramp in throat, at inner side of cervical vertebræ; with difficulty of swallowing.

*Heat and cold.*—Pains relieved by heat, worse from cold.

*Moving the head.*—Stitching and tensive pain in swollen parotid gland (the right).

*Turning the head.*—Aggravation of soreness of trapezius muscles, and sensitiveness of neck to the touch.

*Pressure and touch.*—Pain or bruised feeling in swollen submaxillary glands.

*Morning.*—Sitting up in bed; *painful jerk from throat to pit of the stomach.*

*Morning.*—When rising; sweat on the forehead, weakness and qualmishness, hawking up phlegm.

*Afternoon.*—Heat in the throat, and fullness and pressure in the head; paroxysms of pain in throat attended with lachrymation.

*Evening.*—Scraping in throat when swallowing; sensation of a plug in throat; *muscles of deglutition seem paralyzed* for several *evenings.*

Just before going to sleep, dryness and contracted sensation in the throat; dryness not relieved by drinking.

---

*NOTE.—Dr. Allen's *Encyclopædia* modifies this extract from *Chronic Diseases*, probably from clinical observation, substituting words "whooping and choking" for "short and hacking," and words "swallowing saliva" for "swallowing food," as here given. B. L. B. B.

*Night.*—Frequent waking, with great accumulation of mucus in the throat; distressing dreams.

CONCOMITANTS.

*Mental.*—Sadness, melancholy, anxiety, irritability, sensitiveness.

On rising in the morning, weakness, sweat on the forehead, and qualmishness; dullness of the head; heat over the whole body; pulse 108, followed by a stupid condition, in which he did not know whether he was asleep or awake. With heat of the whole body, thirst, and burning of the eyes. Offensive breath, foul taste as of old catarrh; sour taste. Extreme nervous restlessness; great faintness, with heat, then coldness; languor, feeling of prostration; great weakness; frequent trembling of the whole body; ebullition of blood in the whole body; drawing pain all over, even in the bones of the arms. Very sensitive to cold, cold air disagreeable. Symptoms more severe on the left side, generally better after supper; better in the open air, from active exercise, except riding on horseback. Characteristic skin eruptions, are red-wine-colored spots, red itching herpetic spots on both sides of the neck; vesicles on the upper lip; itching blisters and blotches on face, hands, and feet; blisters form easily on the heel.

*Cured by Sepia.*—Naso-pharyngeal inflammation and catarrh, diphtheria, faucial and laryngeal laryngitis, pustular tonsilitis. (Sepia $^{800}$; Epithelioma of lower lip, Dr. Dunham.)

Authorities: *Hahnemann's Chronic Diseases, Allen's Encyclopœdia,* mostly quoted verbatim.

B. L. B. Baylies.

